# Brachial Neuritis With Phrenic Nerve Involvement in a Patient With a Possible Connective Tissue Disease

**DOI:** 10.1177/2324709614535203

**Published:** 2014-05-09

**Authors:** Meera Subash, Gaurav Patel, John Welker, Kenneth Nugent

**Affiliations:** 1 Texas Tech University Health Sciences Center, Lubbock, TX, USA

**Keywords:** brachial neuritis, neuralgic amyotrophy, bilateral diaphragmatic paralysis, bilateral phrenic nerve injury, mixed connective tissue disease, immune injury

## Abstract

*Background*. Brachial neuritis (BN) is a rare inflammatory condition of peripheral nerves, usually involving the cervicobrachial plexus. These patients present with sudden onset of shoulder and arm pain that evolves into muscle weakness and atrophy.. *Case Report*. A 33-year-old woman presented with a 1-month history of diffuse pain in her thorax. She had no trauma or inciting incident prior to the onset of this pain and was initially treated for muscle spasms. The patient was seen in the emergency room multiple times and was treated with several courses of antibiotics for pneumonia on the basis of clinical symptoms and abnormal x-rays. The pleuritic chest pain persisted for at least 4 months, and the patient was eventually admitted for worsening pain and dyspnea. On physical examination, crackles were heard at both lung bases, and chest inspection revealed increased expansion in the upper thorax but poor expansion of the lower thorax and mild paradoxical respiration. “Sniff” test revealed no motion of the left hemidiaphragm and reduced motion on the right hemidiaphragm. Her computed tomography scan revealed bilateral atelectasis, more severe at the left base. She reported no symptoms involving her joints or skin or abdomen. Her presentation and clinical course are best explained by BN with a bilateral diaphragmatic weakness. However, she had a positive ANA, RF, anti-RNP antibody, and anti SS-A. *Conclusion*. Patients with BN can present with diffuse thoracic pain, pleuritic chest pain, and diaphragmatic weakness. Our patient may represent a case of connective tissue disease presenting with brachial plexus neuritis.

## Introduction

Brachial neuritis (BN) or neuralgic amyotrophy, or Parsonage-Turner syndrome, is a rare idiopathic neuropathy that can present with a variety of lower motor neuron symptoms. These symptoms often occur abruptly after surgery, trauma, illness, or immune system stress and include prominent pain and neurological and/or musculoskeletal symptoms.^1,2^ The etiology of this condition is unknown; it has been linked to autoimmune and inflammatory processes that infiltrate nerve fibers that originate in the brachial plexus. There is also an inherited form of this disorder with autosomal dominant inheritance that has been linked to mutations in the septin gene SEPT9 on chromosome 17q. We report a patient with persistent pleuritic pain, bilateral phrenic nerve weakness, and atelectasis secondary to BN who also had positive serological tests for connective tissue disease.

## Case

Our patient is a 33-year-old African American woman with a past history of hypertension and hypothyroidism who had repeated emergency room visits for pleuritic chest pain and dyspnea. On her initial presentation to the emergency room, she reported intermittent pleuritic pain in the right anterior region of her chest. The pain was described as 10/10 in severity, increased with both inspiration and palpation of the chest wall, and radiated into her back. Her pain was aggravated with walking and while lying flat to the point that she was sleeping on 3 or more pillows. The patient also reported a mild productive cough with clear-yellow sputum and some blood. She had no dysphagia. On chest x-ray, she had bilateral lower lobe and lingular airspace consolidation prompting the diagnosis of community-acquired pneumonia. The patient was treated with a 7-day course of antibiotics and antitussive medications. Over the next 4 months, the patient returned to the emergency room 5 times with similar complaints of pleuritic chest pain and dyspnea. She also noted pain in the neck, both arms, and upper back during this 4-month course. At her last emergency department visit, the patient still complained about left arm pain associated with paresthesias. Repeat imaging of her chest continued to show lower lobe consolidation/atelectasis. Since her symptoms had failed to improve with multiple courses of antibiotics, she was admitted to the internal medicine service for further evaluation.

The patient had a past history of hypertension and hypothyroidism. She had no history of recent surgery, trauma, infection, or radiation. Her family history was positive for hypertension in her father and lung cancer in her grandmother but negative for connective tissue disease. She was married, worked as a nurse’s aide, had an occasional alcoholic drink, and smoked 1 cigarette per day.

Initial vital signs included blood pressure 131/87 mm Hg, pulse rate 109 beats per minute, respiratory rate 18 breaths per minute, temperature 99.4°F, and oxygen saturation 98% on room air. Initial complete blood count included white blood cells 5300/µL, hemoglobin 9.5 gm/dL, and platelet count 278 000/µL. The initial complete metabolic panel was normal except for a low K^+^ (3.4 mmol/L). Creatine kinase was 29 IU/L (normal = 26-308 IU/L). The thyroid-stimulating hormone level was normal off thyroid replacement therapy. The erythrocyte sedimentation rate (ESR) was121 mm/h (normal = 0-20), and the C-reactive protein (CRP) level was 8.2 mg/dL (normal = 0-0.5). Other laboratory tests included a positive ANA using the BioPlex 2200 ANA screen (BioRad Laboratories, Inc, Hercules, CA). Additional testing included a positive chromatin with an Antibody Index (AI) of 2.9 (normal = 0.2-1.0). Anti-double stranded DNA and anti-Smith antibodies were negative. SS-A antibody was 8 AI (normal = 0.2 to1.0); the SS-B antibody was negative. The rheumatoid factor (RF) was 20 IU/mL (normal < 14), and the cyclic citrulline peptide was negative. The anti-RNP antibody was >8.0 AI (normal = 0.2-1.0). Blood cultures were negative. Her echocardiogram revealed a small atrial septal defect, mild pulmonary hypertension with right ventricular systolic pressure of 35 to 40 mm Hg, and an ejection fraction of 65% to 69%. The pulmonology medicine consultant noted that the patient had poor inspiratory expansion of the lower thorax, paradoxical movement of her abdomen, and bilateral basilar crackles. Her voice was normal. Neurosurgery was consulted and documented normal strength in the arms and legs, normal sensation to light touch, normal proprioception, normal reflexes, and normal coordination. Magnetic resonance imaging (MRI) of the cervical spine showed only some mild degenerative changes and no evidence of focal lesions that could cause radiculopathy. A fluoroscopic “sniff” test showed no movement of the left diaphragm and reduced movement on the right, consistent with bilateral phrenic nerve involvement and respiratory muscle weakness. She was subsequently prescribed muscle relaxants, pulmonary hygiene with a vibratory therapy system, incentive spirometry, and pain medication, including indomethacin for symptomatic relief. Her pain and dyspnea improved somewhat, and she was discharged home.

While in the hospital, she was started on incentive spirometry and an albuterol inhaler 1 to 2 puffs every 6 to 8 hours. Her inhalation volume on incentive spirometry was 500 mL initially in the hospital, 1000 mL after 1 month, and 1500 mL after 2 months. Pulmonary function tests done 2 months after the initial hospitalization revealed a forced expiratory volume in 1 second (FEV_1_) 1.4 L (43.9% predicted), forced vital capacity (FVC) 1.9 L (47.3% predicted), FEV_1_/FVC ratio 0.77, total lung capacity 3.7 (60.8% predicted), a normal residual volume 1.7 L (105.2% predicted), and a severely reduced diffusing capacity for carbon monoxide (36.4% predicted) that partially corrected with adjustment for alveolar volume (67.7% predicted). Pulmonary function tests were consistent with a severe restrictive ventilatory defect with a reduced diffusion capacity.

Two months later the patient described her chest pain as markedly improved compared to her first admission. She reported her pain at rest as 3/10, mild in intensity, in the lower part of her rib cage but nonradiating, and aggravated with daily routine activities. She denied fever, chills, nausea, vomiting, and joint pain in her hands but still reported mild shortness of breath at rest. She had had several visits to a rheumatologist who started her on a regimen of hydroxychloroquine, methotrexate, and low-dose prednisone (5 mg daily) for suspected lupus and seropositive rheumatoid arthritis and eventually added leflunomide and etanercept. The patient noted some symptomatic relief on this new regimen but now noticed her fingers turning white in cold temperatures. Over a 1-year follow-up period she had persistent pain and some joint tenderness on palpation but no synovitis. Repeat ANA was positive 3 months later, and a repeat ESR was 95 mm/h 6 months later. Skeletal x-rays done 6 months after her initial presentation with chest pain showed no evidence of rheumatoid arthritis.

## Discussion

Brachial plexus neuritis usually presents with a sudden onset of sharp, severe pain in the shoulder and arm that lasts for 2 to 4 weeks. The classic presentation involves the upper brachial plexus (C5-C6) with the rapid onset of paresis and atrophy of upper extremity muscles. These patients can have motor, sensory, and autonomic symptoms. An “extended” syndrome with nerve involvement outside the brachial plexus also occurs, and this presentation can involve the lumbosacral plexus, the phrenic nerve, and/or the recurrent laryngeal nerve. Bilateral phrenic nerve involvement occurs in less than 1% of cases.^1^ In most cases, BN is a self-limited illness, and patients have full restoration of function within a few weeks to months. However, some studies report that 25% of patients are not able to return to work after 3 years. The use of corticosteroids is controversial, and no randomized controlled trials have confirmed their efficacy.^1^ A retrospective study reported by van Eijk et al suggested that oral prednisolone was effective but only 12% of treated patients had fully recovered within 1 year.^3^ Our patient had persistent symptoms in the chest, arms, and shoulders for 4 months; had bilateral phrenic nerve involvement; had no apparent trigger; and did not improve significantly with medications for rheumatoid arthritis, including corticosteroids and disease modifying agents. Her pleuritic pain is likely explained by involvement of the long thoracic nerve that supplies the serratus anterior muscle. This muscle attaches to the upper 8th to 9th ribs. Patients with BN often have pain at insertion points of paretic muscles, and this would explain her pain on palpation and inspiration.^1,2^ Our patient did not have an EMG/nerve conduction study, which can show reduced nerve conduction velocity and acute denervation in BN patients. She also did not have MRI using a brachial plexus protocol, which can show nerve involvement with hyperintense signals on short tau inversion recovery sequences and neurogenic muscle edema on T2 weighted fast spin echo images.^4,5^ These studies were deferred since her predominant nerve involvement involved the phrenic nerves, and diaphragmatic weakness was established with fluoroscopic studies. The fluoroscopic sniff test involves the measurement of diaphragmatic movement during a quick inspiratory effort (a sniff) and is particularly useful for unilateral paralysis. Her clinical course during a 1-year follow-up did not suggest an occult malignancy with a paraneoplastic syndrome.

Phrenic nerve involvement with BN occurs infrequently.^6-10^ This diagnosis requires a complex synthesis of a detailed history of persistent shoulder, back, and/or neck pain and possible other peripheral nerve palsies along with chest radiographic findings and fluoroscopic results. Computed tomography scans of the cervical and thoracic spine are required to rule out myelopathy and nerve root compression. Electrophysiological tests, including EMGs, can help distinguish between axonal injury and demyelination and help identify other diagnostic possibilities.^1,2^ Bilateral involvement, as in our patient’s case, causes a restrictive defect on pulmonary function tests, and these patients should have pulmonary function tests, including vital capacity measurements in both the supine and upright positions. Patients with “idiopathic” phrenic nerve injury should be reviewed for the possibly of undiagnosed BN.^11^

Patients with BN have patchy axonal degeneration of nerves. Peripheral nerve biopsies have revealed perivascular T-cell infiltrates and B-lymphocyte germinal centers.^1^ The pathogenesis likely involves an underlying unknown predisposition, autoimmune factors, and biomechanical factors. No genetic abnormalities have been identified in patients with idiopathic BN. Some patients (approximately 55%) with hereditary BN have mutations in the SEPT9 gene, which produce abnormal protein filaments that have an uncertain role in pathogenesis. More than 50% of patients with BN have an immune stressor prior to onset. These include infection, vaccination, surgery, pregnancy, and immunotherapy. Vriesendorp et al studied 3 patients with BN and detected antibodies to peripheral nerve myelin in them. One also had elevated soluble terminal complement activation products.^12^ Finally, microtrauma secondary to repeated movement of the hypermobile shoulder at work or with routine activities may contribute to injury and/or help explain the chronicity of symptoms.

The unique features in our case include a neuropathic disorder presenting with pneumonia-like symptoms and pleuritic chest pain that actually reflected respiratory muscle weakness causing atelectasis. Her high ESR, high CRP, positive RF level, positive ANA, positive anti-SS-A, and positive anti-RNP antibodies suggest that a connective tissue disorder might explain her disease. Patients with connective tissue disorders can have peripheral nerve pathologies, including mononeuritis multiplex, distal symmetric neuropathy, fulminant motor neuropathy, compression neuropathy, and sensory neuropathy.^13^,^14^ The incidence of pleuropulmonary manifestations in patients with mixed connective tissue disease (MCTD) ranges from 20% to 85%.^15,16^. The respiratory presentations can include interstitial lung disease, pulmonary fibrosis, pleural effusion, and/or pulmonary hypertension. Other manifestations may include vasculitis, thromboembolism, infections, pulmonary nodules, mediastinal lymphadenopathy, and respiratory muscle weakness.^16^ Diaphragmatic involvement in these patients is unusual.^16^ However, she did not have the typical symptoms of MCTD, namely, the combined features of systemic lupus erythematosus (SLE), scleroderma, or rheumatoid arthritis. Specifically, she did not have synovitis, myositis, or swollen fingers and did not meet the criteria proposed by Kahn.^15^ Patients with SLE can have acute pneumonitis, interstitial lung disease, vanishing lung syndrome, and peripheral neuritis and can present with brachial plexus neuropathy.^17^ She did not meet the diagnostic criteria for SLE and, in particular, did not have skin involvement. She also did not meet the criteria for either scleroderma or rheumatoid arthritis and did not have the pulmonary involvement typically associated with these 2 diagnoses. However, she did have persistent immunologic reactivity and persistent elevation of her ESR and CRP; it is possible that she has an undifferentiated connective tissue disease. These patients often have arthralgias, arthritis, Raynaud’s phenomenon, leukopenia, anemia, sicca symptoms, and a positive ANA (60% to 100%).^18^ Some patients with an undifferentiated connective disease evolve into a definite connective tissue disease, some remit, and some have persistent symptoms but no evolution into a clear-cut diagnosis. They usually respond to low doses of prednisone. It is possible our patient’s clinical syndrome will evolve into a definite connective tissue disease and that her abnormal serological tests represent an underlying immune process contributing to her nerve injury. However, her autoimmune tests present a complex pattern, and she has not remitted during a 1-year follow-up on a complex drug regimen. Her ANA screen test remained positive over the initial 4-month follow-up.

## Conclusion

Our patient presented with pleuritic chest pain and dyspnea that actually reflected BN involving the phrenic nerves. Her positive ANA, positive anti-SSA, and positive anti-RNP antibodies suggest that she has an underlying mixed connective tissue disease, but she did not meet standard criteria for these diagnoses over a 1-year period of observation. Consequently, it is difficult to decide whether she has a connective tissue disorder with neuropathy or a primary neuropathic disorder with nonspecific serological markers. Rheumatologists and pulmonary physicians need to consider these alternatives when evaluating these patients and provide follow-up information on patients like ours.

## Key Statements

1. Patients with BN can have complex pain syndromes and clinical presentations, including phrenic nerve injury. Some patients have prolonged disability.
2. The pathogenesis likely involves unknown underlying predispositions to nerve injury and microtrauma related to the hypermobility of the shoulder. Immune responses also contribute to the injury.
3. Our case had multiple serological responses associated with connective tissue disease, which might suggest that immune mediated inflammation contributed to her nerve injury.
4. Patients with BN need more immunological evaluation at presentation and during the course of their illness.
